# Everybody's Page

**Published:** 1892-07-23

**Authors:** 


### Everybody's Page.

M. Pasteur's illness has been much exaggerated, and his life has not been endangered.

Literature is to hold a conspicuous position in the World's Fair; copies of the works of every Colorado author are to be exhibited in the Colorado State building. This is a harmless exhibit, which will no doubt delight the heart and tickle the vanity of many an obscure author whose name would otherwise be unknown to fame.

It is well known that in some districts quinine by itself is unable to combat the evil effects of malaria. Taken in combination with eucalyptus oil, it is asserted to give complete immunity. A writer in *Mission Life and Work*, a small paper publishsd at Blantyre, a Scottish mission near Lake Nyasa, in Central Africa, states that twelve grains of quinine, with five drops of eucalyptus oil, were taken every third day by a party during a journey lasting three weeks, from Quilimane, at the mouth of the Zambesi, up into the Lake Country, and that no fever developed at the time or afterwards.

In a small pamphlet recently issued, the Canadian High Commissioner draws attention to the question of the immigration of children. There is still room for the encouragement of the movement under proper supervision. No children are allowed to be sent out except under the care of individuals or societies which have homes in the United Kingdom as well as in Canada, and the societies in Canada are obliged to return children who, from various causes, are unsuitable and do not turn out satisfactorily, while in every case a medical certificate is required as to the health and physical condition of the child. Objections, no doubt, can be made to the immigration of children, but under a scheme so well guarded as that advocated by the High Commissioner much good work is possible.

Jamaica has never held a very enviable reputation for climate, but Lord Brassey's assertion that it is only "on the loftiest slopes of the Blue Mountains" that "a limited area may perhaps be found where a northern race may enjoy a suitable climate," has called forth a vigorous protest from Mr. Frederic Bernal, whose twelve months' experience on a cattle estate 1,700 feet above the level of the sea leads him to a different opinion. He was able to attend to his work all day out of doors "without suffering any inconvenience from the heat." The belief that Jamaica's climate is not fitted for white men is apparently one without solid foundation, provided judgment is shown in the selection of farms, but it has undoubtedly existed very generally, to the detriment of the colony.

How powerful association is for good or evil is shown in a remarkable manner in the case of a school which has come under the notice of the Government Inspector for the Oxford district. This school a few years ago was dirty both within and without; the children were untidy and in keeping with their surroundings, ill-mannered and noisy, and the walls were defaced with writings and pictures inartistic, and sometimes worse. The vicar, determined, if possible, to put an end to such an undesirable state of affairs, offered to defray the cost of beautifying the interior if the building was thoroughly cleansed. This was done with the most satisfactory result. The personal appearance of the children was soon brought into harmony with their surroundings, and their manners were altered for the better. There is little doubt that more attention might be given to the inculcation of manners amongst the rising generation of Board school children. Incivility and want of respect for their neighbours are too often taken as a sign of independence.

A laudable effort is being made by the National Temperance Café Association to combine amusement with temperance at music-halls in some of the thickly-populated districts of London. The profits to be made out of temperance entertainments are, unfortunately, small compared with those where intoxicating drinks are sold, but the attempt deserves success.

Officialdom cannot be carried to greater excess, it would seem, at any future date, than is already the case in the United States. A contributor to *The Century Magazine* relates that in one small town every man, woman, and child (above ten years of age) holds office, with the exception of a few unambitious and unenterprising people. An eloquent comment on the cheapness and blessings of democracy.

An agitation is on foot in Melbourne urging the Government to establish a female labour bureau, with a view to enabling women who work for their own living to secure employment. At the present time there is much distress there, and many women who have seen better days are in such trouble that they cannot pay a fee to the labour office. It appears that the Chinese are rapidly monopolising laundry work, and it has been suggested that the public should not send their washing to laundries manned by Chinese; but the public will probably go to the cheapest market for their washing as well as for other goods, and will ignore the distress of their fellow countrymen.

We have been assured on the highest authority that there is no known antidote in India to the poison of venomous snakes, but in Australia the natives are said to use a very simple remedy, if bitten in the arm or leg, which is a "safe cure" for the most venemous snake bites. The process is as follows: A piece of human hair string, which is made up as strong and as fine as the best whip-cord, is tied as tightly as possible three or four inches above the region of the bite; then a circle round the bite is cut with a sharp stone knife about an eighth of an inch deep, and a quarter of an inch from the two fang punctures; when this is done the native slits the largest vein below the bite so as to let as much blood as possible out of the limb below the string, and keeps a stream of water running on the limb just above the affected part, rubbing the limb down all the time as hard as possible. This rubbing is kept up for about twenty minutes, till every drop of blood seems to be got out of the wounded portion. When this is accomplished. the slit vein is twitched up with a piece of sharp thin wood, some dirt is dabbed on the wound and the string undone, and Richard is himself again.

A new and hitherto unsuspected danger threatens humanity from the common bug, according to the recent investigations of a French savant. This pest, deservedly detested by clean persons of all nations, has been proved by this doctor to be the medium of transmitting tuberculosis in its most virulent form. Now, if this be so, cannot the fact be utilised as a proof of need for increased attention to the sanitation of the houses of the poor? All district nurses could furnish anecdotes of the horrible army of crawling creatures which parade on walls and appear from old woodwork in many a miserable tenement. The human lodgers are either absolutely indifferent to their bites, or else quietly accept them as one of their ordinary trials, one of the small evils to which their "poverty but not their will consents." If further experiments confirm the accuracy of these investigations, surely the presence of bugs in a dwelling-house might be judged as clear proof that absolute and thorough fumigation alone could obviate all the possible risks of infection. If these insects carry tuberculosis, why may not other diseases have been unsuspectingly transmitted by similar means?

